# Reduction-Triggered Paclitaxel Release Nano-Hybrid System Based on Core-Crosslinked Polymer Dots with a pH-Responsive Shell-Cleavable Colorimetric Biosensor

**DOI:** 10.3390/ijms20215368

**Published:** 2019-10-28

**Authors:** Seul Gi Kim, Benny Ryplida, Pham Thi My Phuong, Hyun Jeong Won, Gibaek Lee, Suk Ho Bhang, Sung Young Park

**Affiliations:** 1 Department of Chemical and Biological Engineering, Korea National University of Transportation, Chungju 380-702, Koreadnjsswh@ut.ac.kr (H.J.W.); glee@ut.ac.kr (G.L.); 2 Department of Green Bio Engineering, Korea National University of Transportation, Chungju 380-702, Korea; ryplida@gmail.com (B.R.); ptmp.2411@gmail.com (P.T.M.P.); 3 School of Chemical Engineering, Sungkyunkwan University, Suwon 16419, Korea; 4 Department of IT Convergence, Korea National University of Transportation, Chungju 380-702, Korea

**Keywords:** nano-hybrid matrix, polymer dots, controllable drug release, pH-sensitive, redox-responsive

## Abstract

Herein, we describe the fabrication and characterization of carbonized disulfide core-crosslinked polymer dots with pH-cleavable colorimetric nanosensors, based on diol dye-conjugated fluorescent polymer dots (L-PD), for reduction-triggered paclitaxel (PTX) release during fluorescence imaging-guided chemotherapy of tumors. L-PD were loaded with PTX (PTX loaded L-PD), via π–π stackings or hydrophobic interactions, for selective theragnosis by enhanced release of PTX after the cleavage of disulfide bonds by high concentration of glutathione (GSH) in a tumor. The nano-hybrid system showed fluorescence quenching behavior with less than 2% of PTX released under physiological conditions. However, in a tumor microenvironment, the fluorescence recovered at an acidic-pH, and PTX (approximately 100% of the drug release) was released efficiently out of the matrix by reduction caused by the GSH level in the tumor cells, which improved the effectiveness of the cancer treatment. Therefore, the colorimetric nanosensor showed promising potential in distinguishing between normal and cancerous tissues depending on the surrounding pH and GSH concentrations so that PTX can be selectively delivered into cancer cells for improved cancer diagnosis and chemotherapy.

## 1. Introduction

Drug delivery innovation has been rapidly advancing, but it requires continuous development to improve therapeutic performance. However, current drug delivery nano-based systems are limited by the need for high dosages, the poor availability of drugs at disease sites, and the unwanted side effects [1]. It is therefore necessary to develop controlled drug delivery with the ability to transport drugs effectively and efficiently to tumor sites [2]. Taking into account the fact that the extracellular microenvironment of tumor tissue is acidic at approximately pH 6.5–6.9 as compared to normal tissue (pH 7.2–7.4) [3], a pH-sensitive nanosystem could be beneficial in distinguishing between tumor and healthy tissues to solve the drawbacks in drug delivery. Additionally, tumor cells contain a reducing substance in the form of glutathione (GSH), which has been proved to be at least four times higher than that in the normal cells [4,5]. Considering both facts, a dual pH and redox-sensitive drug delivery system (DDS) may have great potential for the controlled release of drugs for efficient cancer therapy especially hydrophobic drugs [6].

Polymer-based nano-hybrid delivery systems, especially in cancer therapy, have been extensively investigated as a way to achieve temporal and spatial control for targeting drug delivery at disease sites such as lipid based vesicular, micelle-like structure or nanogel-based DDS systems [2,7,8,9,10,11]. However, most of organic compound-based nano-hybrid systems still need to be improved due to a lack of real-time monitoring properties; they also show low drug-loading percentages and poor stability during blood circulation, thus hindering their performance, which remains a noticeable challenge [12]. Recently, sp^2^-riched carbon hybrid nanocomposites such as graphene oxide (GO) and carbon nanotubes (CNTs) were employed as drug carriers for better stability. However, the nano-based system still showed problems, especially during the drug-release phase [13]. The π–π stackings and hydrophobic interactions hindered the release of the drug during delivery, making the controlled release of a hydrophobic drug to the desired tumor site difficult [14]. Therefore, a drug nano-hybrid matrix containing both hydrophobic and hydrophilic sides and that has a structure similar to that of graphene would improve the stability and drug release during delivery [15]. In recent times, fluorescent polymer dots (PD) have been studied owing to their nanoparticle size, easy preparation, desirable biocompatibility, and sensitivity to environmental conditions such as pH, redox potential, light, and temperature after modifications [16,17,18]. PD have been reported to possess a lattice structure similar to that of GO and the ability to form core-shell structures under aqueous conditions due to rich sp^2^ bonds in the core and hydrophilic segments in the shell, which can be easily loaded with hydrophobic drugs. As compared with conventional systems, PD has performed lower systemic toxicity with prolong drug circulation time that also improved drug solubility and targeting ability. These characteristics suggest that PD could act as stable drug carriers with real-time monitoring properties that would meet the criteria for an intelligent, stimuli-responsive drug carrier.

In this study, we designed a dual-sensitive drug delivery system based on a carbonized form of disulfide-crosslinked pluronic-grafted-2-(dimethylamino) ethyl methacrylate-2-hydroxyethyl methacrylate (B/S-Pluronic) to achieve pH- and redox-responsive fluorescence PD (B-PD). These were then conjugated with fluorescent dyes to form L-PD by utilizing the boron–diol interaction to turn the fluorescence “off”, which hypothetically could recover under acidic pH. Next, the L-PD was loaded with paclitaxel (PTX; PTX loaded L-PD) through π–π stacking or hydrophobic interactions for simultaneous diagnosis and cancer therapy by controlled release of PTX after cleaving disulfide bonds by GSH. It is hypothesized that the designed DDS could show fluorescence quenching behavior at physiological pH and low drug release amounts without GSH. Oppositely, the fluorescence could be recovered by tumor acidic environment and PTX could be sustainably released from the matrices in a time manner to start therapeutic procedure responded quickly to reductive GSH concentration in the cancer cell to enhance the therapeutic efficacy of anticancer drugs. Therefore, the distinguishing characteristic of the proposed system may be the selectivity of a PTX loaded L-PD system in response to surrounding pH and GSH concentrations, thus showing its promising potential for an improved cancer diagnosis and therapy.

## 2. Results and Discussion

### 2.1. Synthesis and Characterization of PTX Loaded L-PD

**Scheme 1:**
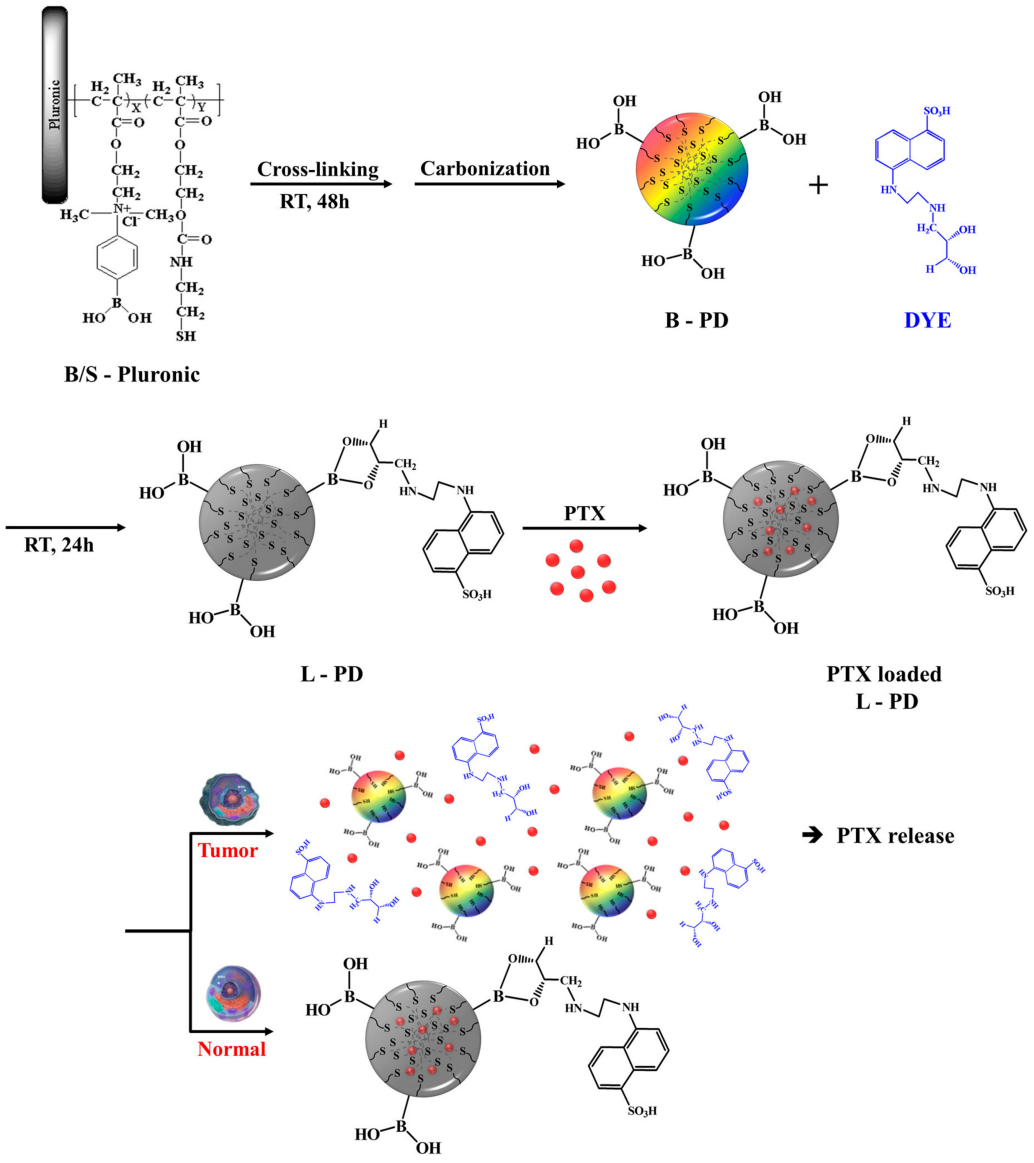
Illustration of the synthesis of dual pH–redox-responsive PTX loaded diol dye-conjugated fluorescent polymer dot (L-PD) nano-hybrid for fluorescent “OFF/ON” and controlled release of PTX in cancer cells.

The synthesis method and principle of simultaneous diagnosis and therapy of PTX loaded L-PD are shown in Scheme 1. The matrices were formed by grafting 2-(dimethylamino) ethyl methacrylate (DMA) and 2-hydroxyethyl methacrylate (HEMA) to Pluronic F-127 to obtain B/S-Pluronic (Scheme S1). To achieve structural stability and redox-responsive functional groups, disulfide bonds (–S–S–) were introduced by allowing the polymer matrices to react with cysteamine; the disulfide-crosslinked polymers spontaneously formed within 48 h. Once B-PD was achieved by carbonization using an oxidative reaction with concentrated sulfuric acid (H_2_SO_4_), B-PD was conjugated with fluorescent dye (DYE) utilizing boron–diol chemistry to achieve fluorescence quenching and pH-sensitive nanoparticles (L-PD). Paclitaxel (PTX) was then loaded into the nano-hybrid matrices by optimizing the π–π stackings or hydrophobic interactions between the aromatic group of PTX and the sp^2^ group of the L-PD to form PTX loaded L-PD [19]. In a tumor environment (acidic pH), it is expected that the disintegration of boronate ester bonds could recover the fluorescence intensity that would subsequently discharge DYE and facilitate further cleavage of disulfide bonds by high concentrations of GSH, releasing PTX to kill cancer cells [20].

**Figure 1:**
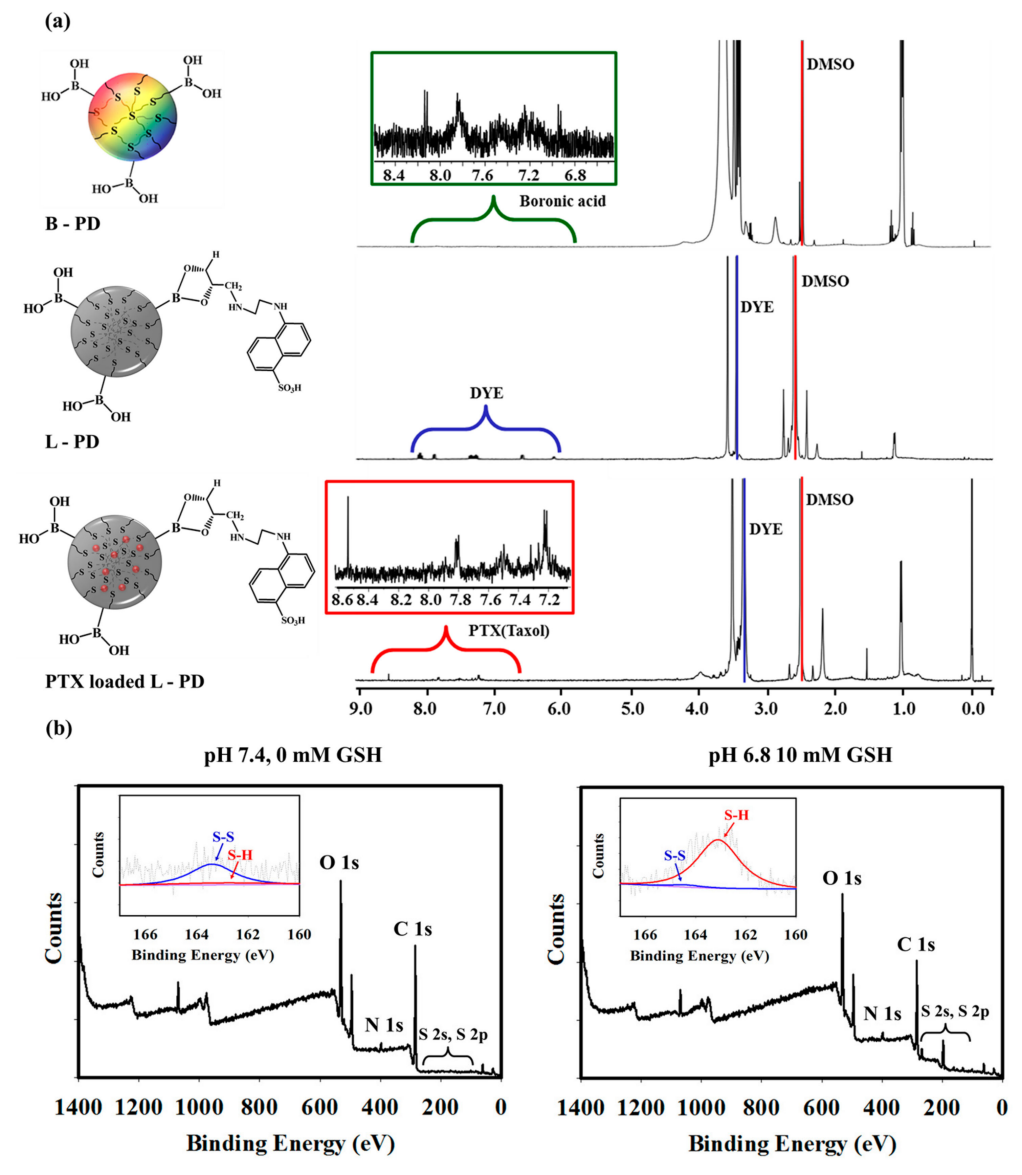
(**a**) ^1^H NMR spectra of redox-responsive fluorescence PD (B-PD), L-PD, and PTX loaded L-PD. (**b**) XPS full survey scan of PTX loaded L-PD at pH 7.4, 0 mM GSH (left) and after treatment at pH 6.8, 10 mM GSH (right).

The chemical structure of the PTX loaded L-PD nanoparticles was analyzed using FT-IR spectra. As shown in Figure S1, the presence of peak at 1020 cm^−1^ of B-PD, which is specific to B–O–H functional groups was observed initially, which then disappear after DYE was introduced, indicating successful conjugation of DYE to B-PD (L-PD) [21]. Moreover, UV-vis spectrophotometry was used to confirm the synthesis results of each nanoparticles. As displayed in Figure S2a, the B-PD displayed broad absorbance peaks between 200–300 nm, which represents a typical absorbance range for *sp^2^* carbon that could act as a fluorophore of the nano-hybrid system [22]. However, the absorbance peak shifted to longer wavelengths after the conjugation with DYE, demonstrating successful attachment of DYE to the B-PD (L-PD). Further confirmation of the conjugation of DYE-PD analysis was obtained using ^1^H-NMR. As shown in Figure 1a, the conjugation of DYE to the B-PD was observed at 7–9 ppm, corresponding to the aromatic peaks of DYE, confirming attachment of DYE onto the PD shells [23]. Additionally, the surface charge properties during synthesis were investigated (Figure S3). The B/S-Pluronic surface charge was 14.8 ± 1.3 mV, which decreased to 7.5 ± 0.9 mV after carbonization owing to the presence of carboxylic groups (B-PD). After DYE was introduced, the zeta potentials slightly shifted to 5.1 ± 0.9 mV due to the −SO_3_ functional groups of DYE molecules (L-PD) and insignificantly dropped to 4.5 ± 0.8 mV after loaded with PTX. As we hypothesized that the disulfide linkages could be cleaved in response to high concentrations of GSH, XPS analysis before and after GSH treatment under different pH conditions was performed (Figure 1b and Figure S6). Without GSH at physiological pH, PTX loaded L-PD showed a strong disulfide (–S–S–) bond peak at 164 eV as compared to the thiol group (–S–H; 162 eV), as displayed in Figure 1b. In contrast, the disulfide bond peak significantly decreased whereas the thiol peak remarkably increased after GSH treatment, confirming the cleavage of –S–S– bonds by GSH [17,24].

**Figure 2:**
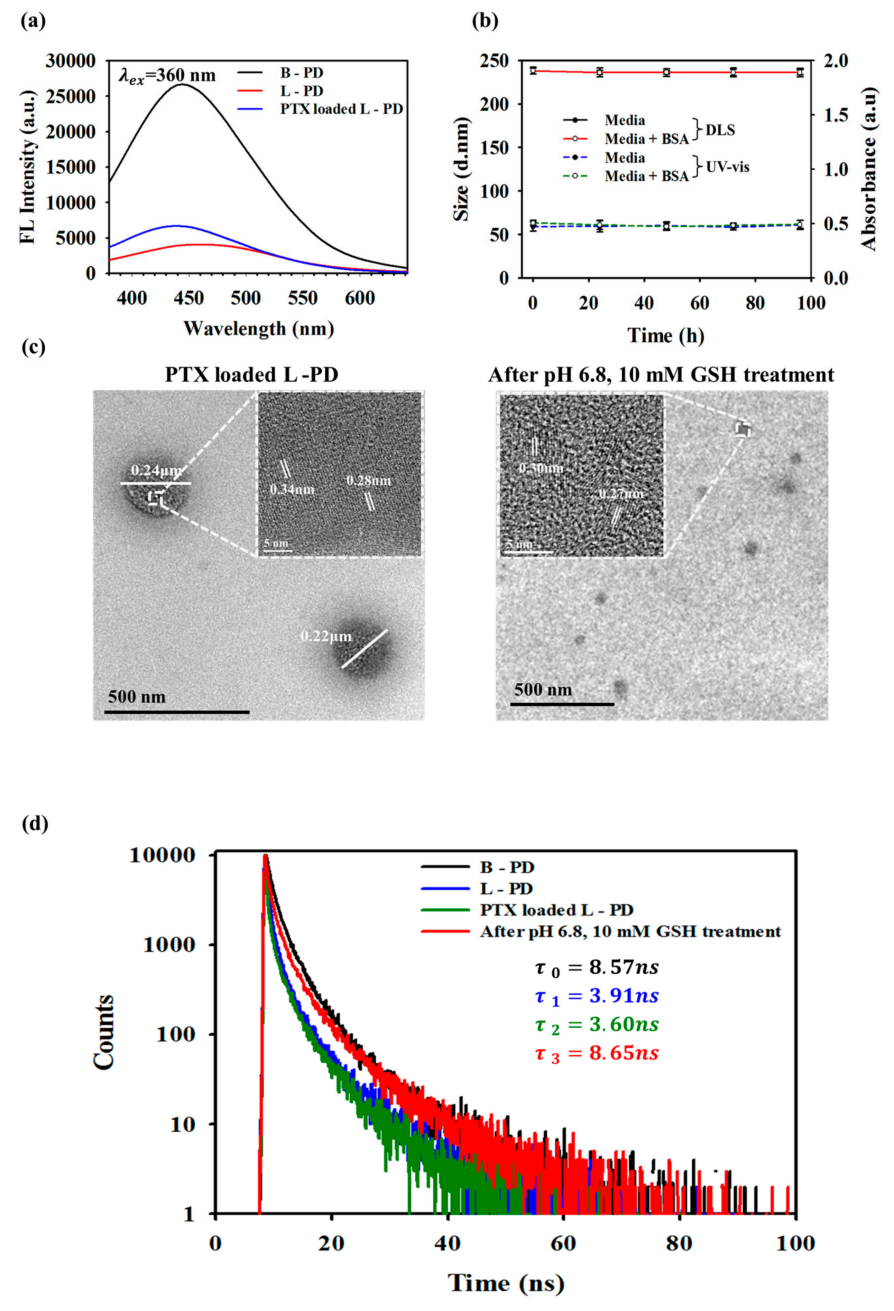
(**a**) Photoluminescence spectra of B-PD, L-PD, and PTX loaded L-PD (excitation λ = 360 nm). (**b**) Time-dependent stability test at 1 mg/mL of PTX loaded L-PD by dynamic light scattering (DLS) and UV-vis absorbance at 260 nm (*n* = 3). (**c**) TEM images of PTX loaded L-PD before and after pH, GSH treatment. (**d**) Fluorescence decay analysis of B-PD, L-PD, and PTX loaded L-PD before and after 10 mM GSH, pH 6.8 treatment at an excitation wavelength of 360 nm.

Real-time monitoring based on the fluorescence OFF/ON nano-hybrid system has promising potential in simultaneous diagnosis and therapy [25,26]; hence, photoluminescence analysis was conducted. As presented in Figure 2a, the fluorescence intensity of the B-PD was high, described as fluorescence “ON”, which then greatly decreased when conjugated with DYE (L-PD), described as fluorescence “OFF” (Figure S2b,c). This decrease was due to the formation of boronate ester bonds, causing the Förster resonance energy transfer (FRET) phenomenon [23,27,28]. Additionally, the fluorescence remained quenched when the PTX was introduced, indicating no fluorescence recovery when the hydrophobic drug was loaded (Figure S2d). In the UV-vis spectra of the PTX loaded L-PD, we observed broad absorbance peaks in the 300–400 nm region for π–π bonding of PD with a PTX absorption peak at 230 nm, which represents loading PTX into the core of the PD (Figure S2a) [29]. Moreover, the average size distribution of nanoparticles in the aqueous state at each step was observed using dynamic light scattering (DLS; Figure S4). The B/S–Pluronic nanoparticles size was 252.06 nm with a polydispersity index (PDI) value at 0.361 suggesting the monodispersity of the samples. After carbonization process, the size of B-PD in aqueous solution become 169.24 nm (PDI = 0.261), which elevated to 238.52 nm (PDI = 0.310) due to the conjugation between the DYE nanoparticles and the polymer dots (boron-ester). Furthermore, the successful loading of PTX was observed after the particle size increased to 245.66 nm (PDI = 0.365) [30]. To investigate the stability of the hydrophobic drug-loaded matrix in biological fluid before reaching the cancer cells, DLS and UV-vis spectra analyses were performed at different times. Figure 2b displays the hydrodynamic sizes and absorption intensities of the PTX loaded L-PD in different solutions over 4 days. No change in size is observed in media and BSA solutions, indicating the high stability of the nano-hybrid system in physiological conditions. TEM images of PTX loaded L-PD before treatment, shown in Figure 2c, exhibited a dot-like morphology with a diameter of around 220 nm and a lattice spacing similar to that of graphene (0.28–0.34 nm) [29]. However, the size decreased to around 45 nm when treated with 10 mM GSH at pH 6.8, which is consistent with the DLS results. The size of the PTX loaded L-PD decreased even more to 37.618 nm in the presence of 10 mM GSH at acidic pH, contributing to the further cleavage of disulfide bonds in the core and DYE disembarkment from the shell [23]. Moreover, the fluorescence decay of the PTX loaded L-PD was analyzed to understand the quenching phenomena caused by DYE nanoparticles. The fluorescence lifetime of the B-PD decreased when DYE was introduced to the nanoparticles (L-PD). Additionally, fluorescence recovery was observed after treatment with pH 6.8 and 10 mM GSH, further confirming the cleavage of boron–diol conjugation, as shown in Figure 2d [31].

**Figure 3:**
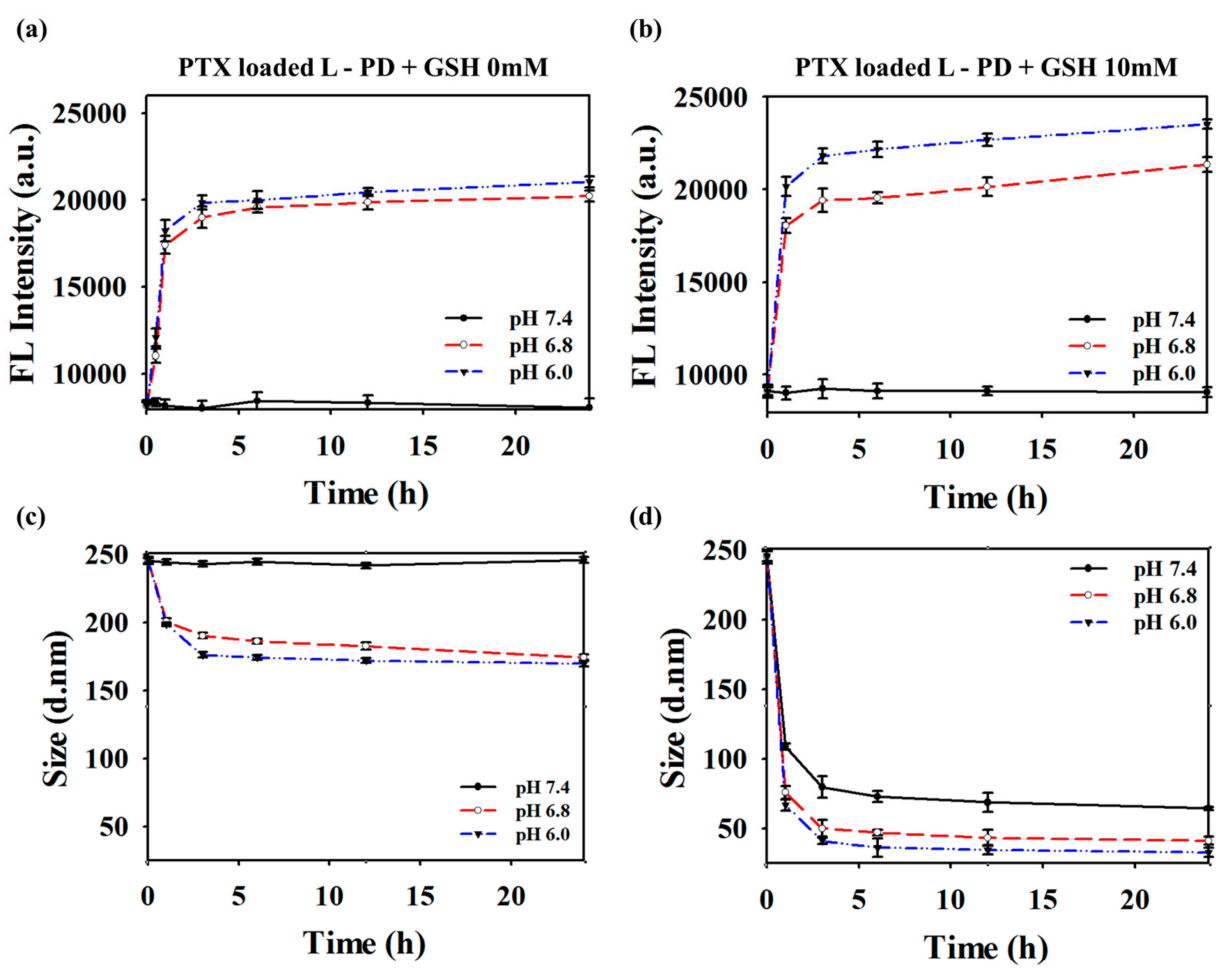
Time-dependent (**a**,**b**) luminescence intensity with excitation at λ = 360 nm and (**c**,**d**) DLS measurements of PTX loaded L-PD at (**a**,**c**) 0 mM GSH and (**b**,**d**) 10 mM GSH treatments in different pH solutions (6.0, 6.8, and 7.4; *n* = 3).

As shown in Figure 3a, for PTX loaded L-PD at physiological pH without GSH, the fluorescent intensity of the PTX loaded L-PD did not show any significant increase due to the non-responsive behavior of the boron–diol conjugation at pH 7.4, even after GSH treatment, confirming the stability of the fluorescent quenching state of the nano-hybrid system. Next, the pH of the solution was varied from 6.0 to 7.4 to study the dot sizes and fluorescent intensity changes. The photoluminescence (PL) signal of the PTX loaded L-PD at λ = 360 nm displays specific enhancement in acidic pH depending on the reaction time [32]. In addition, changes in the PL profile under different pH conditions evaluated after 10 mM treatment shows that all the fluorescence intensities were higher, not just those subjected to acid treatment (Figure 3b). Furthermore, the combination of both acidic pH and 10 mm GSH showed higher fluorescence recovery rather than single treatments. High GSH concentration in cancer cells confirm the possibility of using reduction–oxidation to change the disulfide bonds into thiol bonds, whereas acidic pH values in tumor cells induce hydrolysis of the L-PD boron–diol covalent bond [33]. The simultaneous GSH and acid treatments supported effective splitting of the PTX loaded L-PD nanoparticles to release PTX, resulting in much smaller particles and distinguish fluorescence response. In similar experimental groups and preparation techniques, the hydrodynamic diameter change was observed using DLS measurements over time, as shown in Figure 3c,d. These figures also confirm that high GSH concentrations and low pH values resulted in greater cleavage of the disulfide cross-linked sites to generate smaller particles, whereas no significant change was observed at low levels of GSH [17]. The same phenomenon was observed in the L-PD when treated with different pH values and GSH concentrations (Figure S5).

### 2.2. Stimuli-Responsive Drug Release and Anticancer Efficacy Evaluation of PTX Loaded L-PD

**Figure 4:**
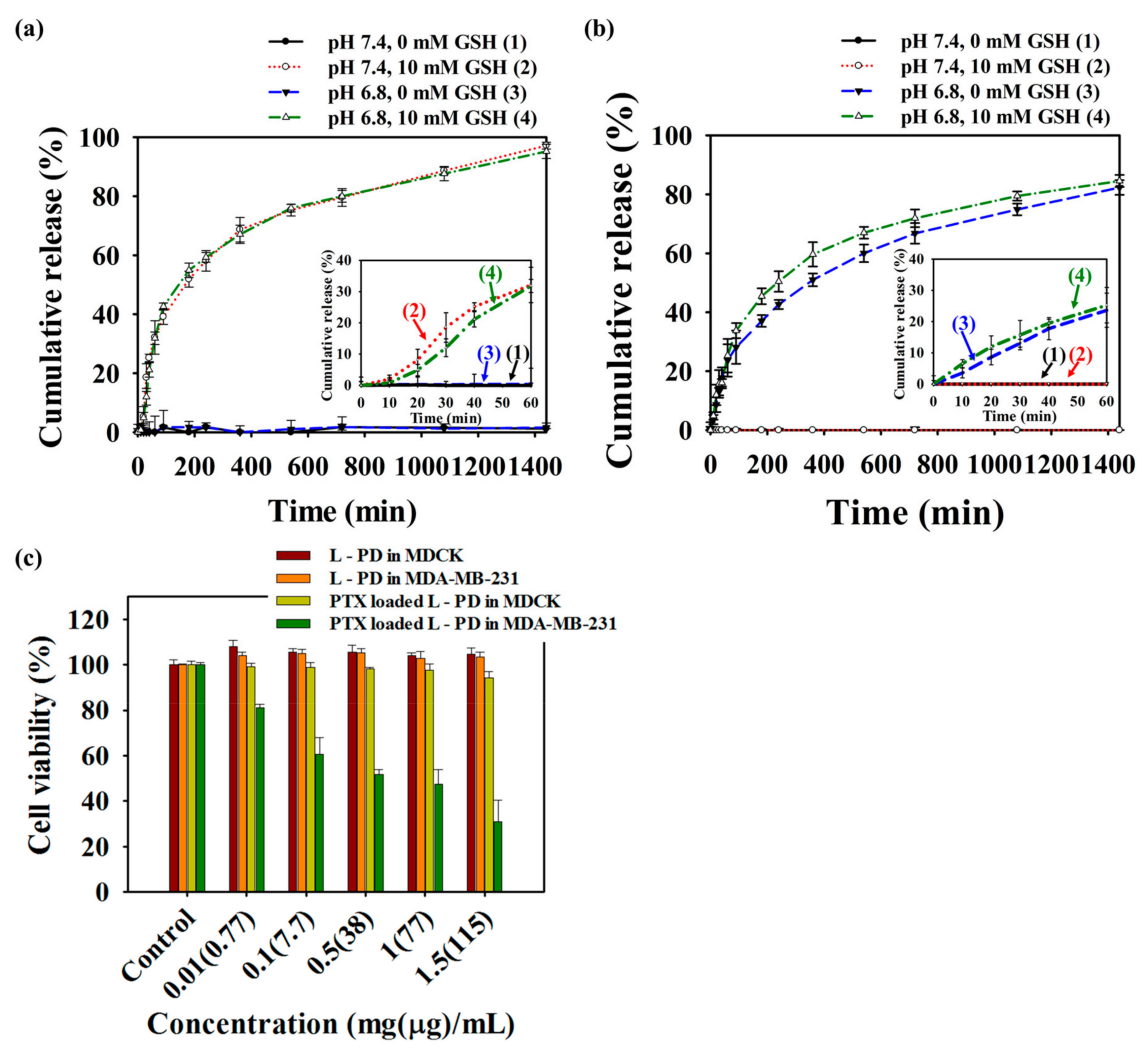
In vitro release profile of (**a**) PTX and (**b**) fluorescent dye (DYE) from PTX loaded L-PD matrices under different concentrations of GSH at physiological (pH 7.4) and tumor (pH 6.8) conditions; inset: first 60 min of release profile. (**c**) Cell viability of Madin-Darby canine kidney (MDCK) and breast cancer cell lines (MDA-MB-231) after treatment with different concentrations of L-PD and PTX loaded L-PD (*n* = 5). Parentheses (μg): PTX concentration in PTX loaded L-PD samples.

The release of PTX in response to acidic and GSH levels in cancer cells were evaluated by UV-vis spectroscopy over time. The loading content (%LC) and encapsulation efficiency (%EE) of PTX were 7.708% and 83.52%, respectively. The successful loading of PTX in the nano-hybrid matrices was dependent on π–π stacking and hydrophobic interactions between PTX and the L-PD. However, the attachment of DYE to the B-PD was determined by the boron–diol conjugation of both types of nanoparticles. The PTX and DYE release profile were conducted at absorbance values 230 nm and 335 nm, respectively, as depicted in Figure 4a,b, respectively. As shown in Figure 4a, the amount of PTX released was less than 5% in the absence of GSH regardless whether pH was at the physiological or the acidic level, suggesting that there was no breakage of L-PD crosslinking and stable hydrophobic interactions. However, burst release was observed after treatment with GSH, which was related to the cleavage of disulfide bonds in the L-PD core. Specifically, 31.8% and 32.1% of PTX amount was released from the PTX loaded L-PD cargo during the first 60 min and 100% after 24 h in the presence of GSH. Disintegration of boron–diol covalent bond of B-PD and DYE was observed in the form of the release profile of DYE (Figure 4b). At pH 7.4 and GSH concentrations of either 0 mM or 10 mM, less than 5% of DYE was released, even after 24 h, suggesting no boron–diol cleavage on the shells of the L-PD. Additionally, we investigated DYE release under acidic pH conditions and GSH concentrations of 0 mM and 10 mM. The results showed a high percentage of DYE, corresponding to the pH effect on the nano-hybrid matrix that induced hydrolysis of the boron–diol covalent bond between DYE and the drug carrier. It should be noted that 23.7% (0 mM GSH) and 25.3% (10 mM GSH) of DYE released from the PTX loaded L-PD matrices in the first 60 min after pH treatment. To understand the efficient delivery, therapeutic performance, and biocompatibility, MTT assay was performed using Madin-Darby canine kidney (MDCK) and breast cancer cell lines (MDA-MB-231) cells. The L-PD nanoparticles demonstrated excellent biocompatibility across a wide range of concentrations from 0.01 to 1.5 mg/mL, as displayed in Figure 4c. Moreover, the viability of the cells remained high for the MDCK cells, even with the presence of PTX, indicating no release or leakage during incubation. However, the number of dead cells remarkably increased for the MDA-MB-231 cells, which had a cell survival of only 37%, which further supported the anticancer effect of the released PTX after cleaving of boron–diol covalent bonds by pH and disulfide bonds by GSH. These phenomena suggest the selectivity of PTX loaded L-PD to the pH environment and GSH concentrations that effectively promoted the therapeutic performance of the nano-hybrid system.

### 2.3. Fluorescence OFF/ON Behavior Based on Confocal Imaging

**Figure 5:**
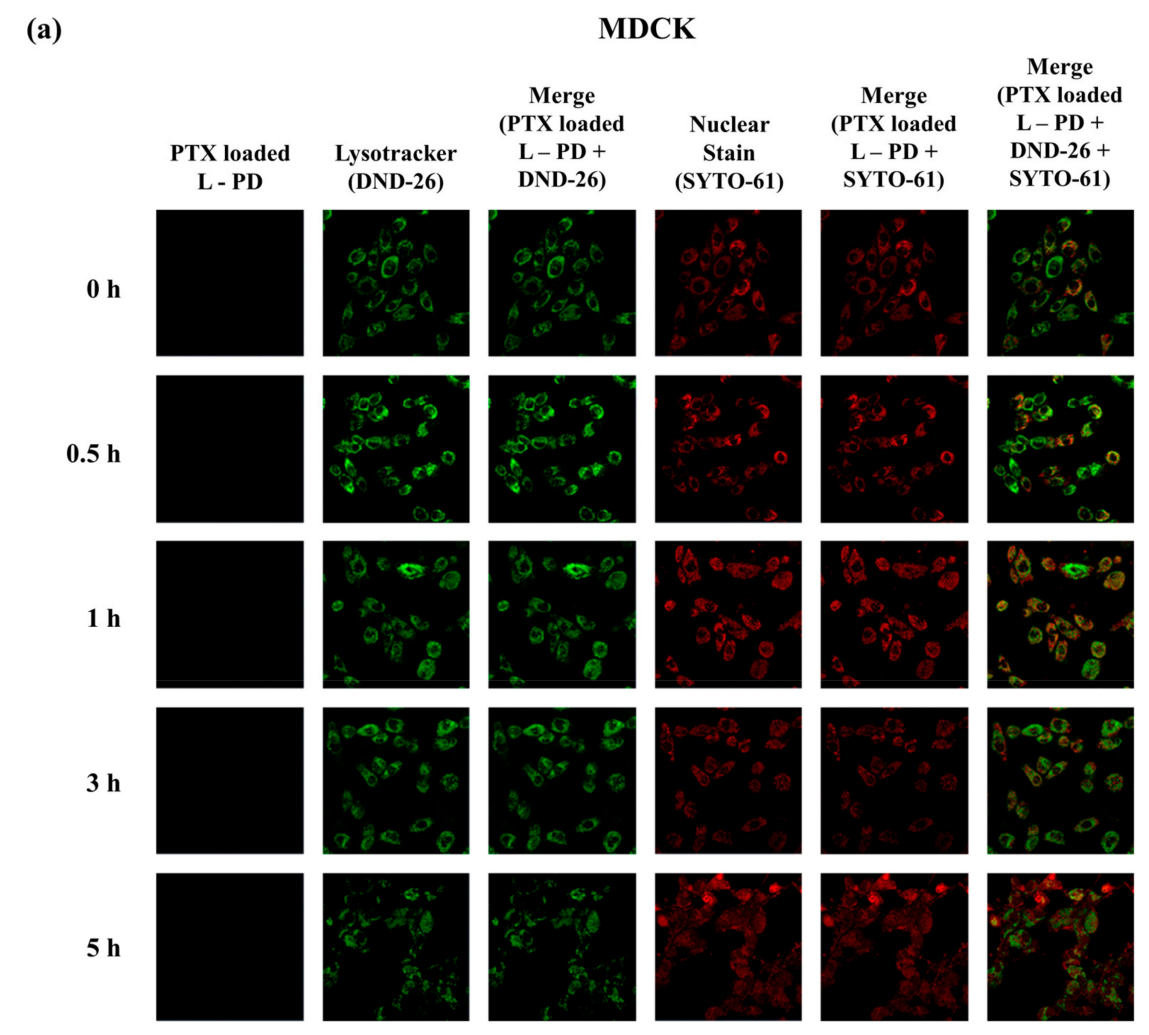

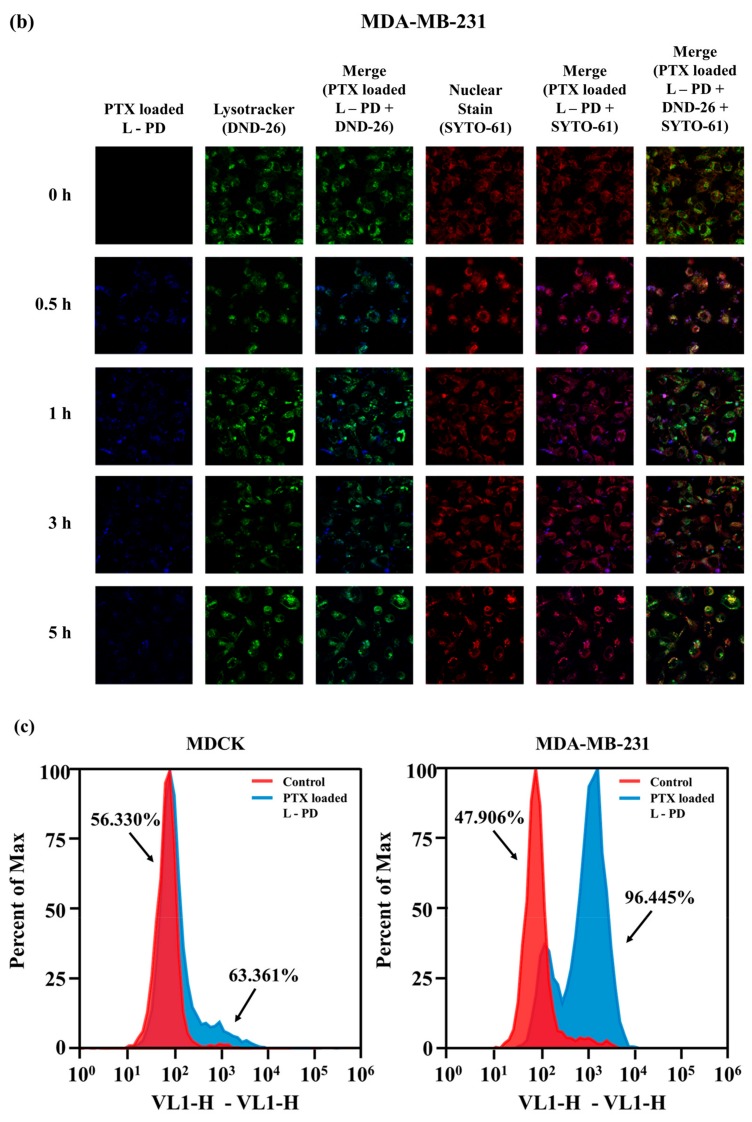
Confocal images of (**a**) MDCK and (**b**) MDA-MB-231 treated with PTX loaded L-PD at different incubation times (0 h, 0.5 h, 1 h, 3 h, and 5 h) stained with a Lysotracker (green) and SYTO 61 (red). (**c**) Flow cytometric (FACS) analysis of in vitro quantitative fluorescence behavior of MDCK and MDA-MB-231 after treatment without (control) and with PTX loaded L-PD. (Scale Bar: 40 µm).

Further investigation to confirm the fluorescence OFF/ON behavior to distinguish normal and cancer conditions was performed using confocal images. Figure 5a displays the images of MDCK cells after incubation with PTX loaded L-PD for different times. No fluorescence signal was revealed from 0–5 h treatment of the MDCK cells, which indicates the stability of PTX loaded L-PD at physiological pH and low concentrations of GSH. In the MDA-MB-231 cells, fluorescence recovery was observed after only 0.5 h, and it remained stable up to 3 h (Figure 5b) due to the hydrolysis of the boron–diol covalent bonds at acidic pH, which increased the fluorescence emission. Moreover, the fluorescence signal diminished after 5 h, corresponding to the endosomal escape of the nanoparticles from the liposomal vesicles. To understand the fluorescence OFF/ON behavior of the nanoparticles by using the color changes of stimuli-responsive PD as a function of the cellular environment of the normal and tumor cells, flow cytometry analysis was conducted. In Figure 5c, MDCK shows an insignificant increase of intensity when treated with PTX loaded L-PD. However, MDA-MB-231 treated with PTX loaded L-PD increased to 96.445%, demonstrating the increase of PD fluorescence intensity in a tumor environment as an effect of the cleavage of DYE-PD boron–diol bonds by the pH condition and disulfide bonds by GSH.

### 2.4. Apoptosis/Necrosis Analysis and Live/Dead Assay

**Figure 6:**
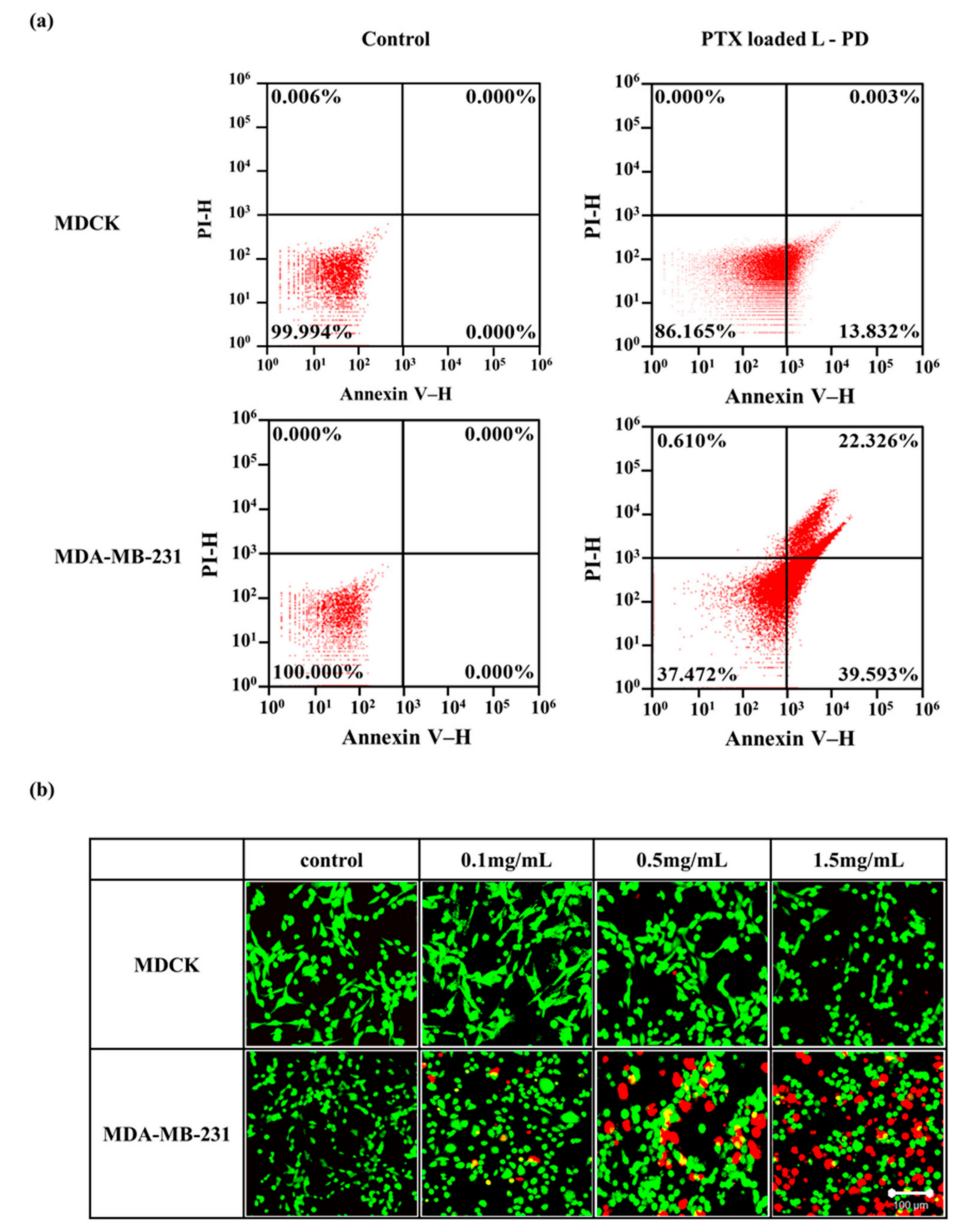
(**a**) Apoptosis assay of MDCK and MDA-MB-231 analyzed with flow cytometry. (**b**) Live and dead cell imaging spectroscopy of MDCK and MDA-MB after treatment with PTX loaded L-PD (Scale Bar: 100 µm).

Further study of PTX loaded L-PD cytotoxicity was proven to be necessary, and therefore flow cytometry was conducted to investigate cellular apoptosis/necrosis using annexin V and propidium iodide (PI). The MDCK cells demonstrated excellent survivability when treated with PTX loaded L-PD as no cells reached late apoptosis and necrosis (Figure 6a). However, a higher percentage of apoptosis cells were counted when the MDA-MB-231 cells were treated with PTX loaded L-PD, corresponding to the release of the drug from the nano-hybrid matrices and the therapeutic effect of PTX. To monitor the cytotoxicity of the PTX loaded L-PD, an in vitro live and dead assay was performed using calcein AM and PI staining. After 3 h incubation with the nanoparticles, confocal images of the MDCK cells (Figure 6b) revealed that most of the cells survived (green), which was in contrast to the MDA-MB-231 cells, which were killed (red), indicating the high cytotoxicity of the PTX loaded L-PD.

## 3. Materials and Methods

### 3.1. Materials and Characterization

Pluronic F-127, 2-(dimethylamino)ethyl methacrylate (DMA), 2-hydroxyethyl methacrylate (HEMA), 4-chlorophenylboronic acid, t-butylperoxybenzoate, sulfuric acid (H_2_SO_4_), cysteamine, ethanol (EtOH), trizma base, toluene, hexane, diethyl ether, paclitaxel (PTX), and glutathione (GSH) were obtained from Sigma-Aldrich, Yongin-si, Gyeonggi-do, Korea. Fetal bovine serum (FBS), penicillin–streptomycin, trypsin-ethylenediaminetetraacetic acid (trypsin-EDTA, 0.03% *w/v*), and Roswell Park Memorial Institute (RPMI)-1640 medium were obtained from Gibco BRL, NY, USA. Phosphate-buffered saline (PBS) pH 7.4 was purchased from Bioneer Corp, Daejeon, Korea. Cell staining dyes (Annexin V, propidium iodide (PI), LysoTracker Green DND-26, and MitoTracker Red CMXRos) were procured from Life Technologies, Oregon, USA. The fluorescent probe (DYE) nanoparticles synthesis process was provided in a previous report [34].

Structural analyses were conducted using ^1^H nuclear magnetic resonance (NMR) using deuterium oxide (Bruker AVANCE 400 MHz spectrometer), UV-vis spectrophotometry (Optizen 2020UV spectrometer, Mecasys Co.), Fourier transform infrared (FTIR) spectra (Thermo Scientific, Madison, WI, USA), and X-ray photoelectron spectroscopy (XPS, Omicrometer ESCALAB apparatus, Taunusstein). The fluorescence behavior was observed using a model L550B luminescence spectrometer (PerkinElmer) and the nanoparticle size was evaluated using a Zetasizer Nano device (Malvern). Cell relating analyses were performed using 3-(4,5-dimethylthiazol-2-Yl)-2,5-diphenyltetrazolium bromide (MTT) methods, flow cytometry (Attune NxT Acoustic Focusing Cytometer, Life Technologies), and confocal microscopy images (LSM510 confocal microscope, Carl Zeiss). Transmission electron microscopy (TEM) imaging was obtained using a JEM-2100F microscope (JEOL). Surface charges were measured by a zeta-potential and particle size analyzer (ELSZ; Otsuka Electronics, Japan).

### 3.2. Synthesis of Carbonized Disulfide-Crosslinked Pluronic-Grafted Poly(DMA), Poly(HEMA) Quaternized Boronic Acid–Cysteamine (B-PD)

The S-Pluronic nanoparticles were synthesized as previously reported [23]. B/S-Pluronic was obtained by dissolving S-Pluronic in ethanol, followed by the addition of 4-chlorophenylboronic acid and stirred for 24 h at 70 °C. The product was then poured into diethyl ether to obtain a purified B/S-Pluronic powder.

To synthesize carbonized disulfide crosslinked B/S-Pluronic, 2 g of the obtained powder (B/S-Pluronic) was dissolved in 1% DMSO in 10 mL of deionized water (DDW) and stirred at 25 °C in air. After 48 h, 10 mL of concentrated sulfuric acid was added and reacted for 1 min, followed by the addition of 185 mL of DDW. The product was then dialyzed (MWCO: 3500 Da) for 1 day, freeze-dried, and was labeled as B-PD. (82% Yield).

### 3.3. Synthesis of the Diol-Conjugated Fluorescent Probe- B-PD (L-PD)

The carbonized disulfide-crosslinked B/S-Pluronic (100 mg) and DYE (0.001 mg/mL) were dissolved in 10 mL of Tris-buffered saline (TBS) at pH 12.0. The mixture was stirred for 24 h at room temperature. The solution was dialyzed (MWCO 1 kDa) against water overnight, freeze-dried, and labeled as L-PD (67% Yield).

### 3.4. Drug Loading into L-PD (PTX Loaded L-PD) and Release Profiles

First, 100 mg of the L-PD in 20 mL of PBS (pH 7.4) and 5 mg of PTX in ethanol were stirred together overnight at 24–25 °C. The sample was then purified by dialysis (MWCO 1 kDa) for 24 h and freeze-dried to achieve PTX loaded L-PD (76% yield). The PTX loading content (%LC) and PTX encapsulation efficiency (%EE) were measured with a UV-vis spectrophotometer at 225 nm and calculated using following equations [24]:

(1) $$\%\mathrm{LC}=\frac{\mathrm{weight}\;\mathrm{of}\;\mathrm{drug}\;\mathrm{into}\;\mathrm{the}\;\mathrm{carrier}}{\mathrm{weight}\;\mathrm{of}\;\mathrm{nanoparticles}}\times 100.$$

(2) $$\%\mathrm{EE}=\frac{\mathrm{weight}\;\mathrm{of}\;\mathrm{PTX}\;\mathrm{loaded}\;\mathrm{in}\;\mathrm{nanoparticles}}{\mathrm{weight}\;\mathrm{of}\;\mathrm{PTX}\;\mathrm{in}\;\mathrm{Feed}}\times 100.$$

The drug release profile was performed at different concentrations of GSH (0, 3, 6, and 10 mM) inside the chamber while soaked in approximately 30 mL of release solutions at pH values of 6.8 and 7.4. The sample was positioned inside a shaking incubator at 37 °C and evaluated with UV-vis spectrophotometry to quantify the release of PTX.

### 3.5. Cytotoxicity Assay

To evaluate the cytotoxicity of the PTX loaded L-PD, MDCK and MDA-MB-231 cells at densities of 1 × 10^5^ cells/mL were cultured in a 37 °C incubator (humidified 5% CO_2_ atmosphere) for 24 h. A stock solution containing the PTX loaded L-PD in RPMI-1640 medium (1.5 mg/mL) was prepared and diluted to 0.01 mg/mL serially. The cell media was replaced with stock solution and incubated overnight. The MTT solution was added after the removal of the stock solution. The cell viability was evaluated by using a microplate reader with an absorbance wavelength of 570 nm [35].

### 3.6. Flow Cytometry

The flow cytometry was conducted by culturing MDCK and MDA-MB-231 cells in six-well plates at densities of 1 × 10^6^ cells/mL for 24 h at 37 °C (5% CO_2_). The medium was then changed with a stock solution (dissolved PTX loaded L-PD in media) followed by incubation at the designated time. The samples were then collected and dissolved with 1% FBS in PBS (pH 7.4) and evaluated using filter at excitation wavelength of 405 nm and an emission wavelength of 440/50 nm.

For apoptosis assays, the cells were directly evaluated after stained with annexin V and PI using an excitation wavelength of 488 nm and emission wavelengths of 530/30 nm and 574/26 nm, respectively.

### 3.7. Confocal Imaging

The time-dependent internalization of the PTX loaded L-PD was investigated using confocal laser-scanning microscope (magnification 20×). First, the cells were cultured then treated with sample (1 mg/mL) in medium (different incubation time). The medium was then removed, washed with PBS (pH 7.4), stained with LysoTracker Green (acidic compartment) for 30 min and SYTO 61 (nucleic acid) for 15 min, and observed using a confocal microscope.

For live/dead cell analysis, cultured MDCK and MDA-MB-231 cells were incubated with PTX loaded L-PD (0.2 mg/mL) for 3 h. Then, the prepared cells were stained with calcein AM and PI and placed under confocal laser-scanning microscope (magnification, 10×) to evaluate the stained cells (live or dead).

## 4. Conclusions

In conclusion, a dual-sensitive fluorescent polymer dots (PD) as a paclitaxel (PTX) drug carrier was successfully developed based on a disulfide bond as core-crosslinked and boron-ester as shell-cleavable parts. The PTX loaded L-PD nano-hybrid system core part utilized disulfide bond as a redox-responsive site and for stabilizing the inner structure, which avoid the leaks of PTX drugs during treatment. Meanwhile, the attachment of DYE on the shell part of PD was beneficial for a pH-sensitive bioimaging probe due to the fluorescence quenching at the alkali condition, which recovered at acidic pH owing to boron-ester hydrolysis. The PTX loaded L-PD demonstrated low fluorescence intensity at physiological pH, which recovered after the pH become acidic. Additionally, the release of PTX, which was expected to show distinguish result during different GSH concentrations, was proven to be the same as hypothesized with burst released detected at high concentration of GSH. Moreover, time dependent MDCK treated PTX loaded L-PD confocal images displayed no fluorescent signal while MDA-MB-231 started to showed a fluorescence signal at 30 min treatment. Hence, this nanosystem represents an improved system for differentiating between normal and tumor sites depending on the surrounding pH and GSH concentrations, which could enable controlled release of drugs with bioimaging abilities, showing its high potential for further applications in cancer diagnosis and treatment.

## Supplementary Materials

Supplementary materials can be found at https://www.mdpi.com/1422-0067/20/21/5368/s1. Scheme S1: Synthesis of B/S Pluronic.; Figure S1: Fourier-transform Infrared (FT-IR) spectra of B-PD, L-PD, and PTX loaded L-PD.; Figure S2: (a) UV–vis spectra of B-PD, L-PD, and PTX loaded L-PD (concentration 1 mg/mL). Photoluminescence spectra of (b) B-PD, (c) L-PD, and (d) PTX loaded L-PD.; Figure S3: Zeta potentials of B/S-Pluronic, B-PD, L-PD, and PTX loaded L-PD in PBS pH 7.4 (*n* = 3).; Figure S4: Average size distribution of of B/S-Pluronic, B-PD, L-PD, and PTX loaded L-PD in PBS solution pH 7.4 (Concentration: 0.5 mg/mL).; Figure S5: (a,b) Luminescence intensity with excitation at λ = 360 nm and (c,d) DLS measurements of L-PD at different GSH concentrations (0 and 10 mm GSH) and pH values (6.0, 6.8, and 7.4) over time (*n* = 3).; Figure S6: XPS narrow scan (C 1s, N 1s, and O 1s) of PTX loaded L-PD at pH 7.4 and 0 mM GSH (a) after treatment at pH 6.8 and 10 mM GSH (b).

## Abbreviations

B-PD	Disulfide-crosslinked Pluronic-grafted poly(DMA), poly(HEMA) quaternized boronic acid–cysteamine
B/S-Pluronic	Pluronic-grafted poly(DMA), poly(HEMA) quaternized boronic acid–cysteamine
BSA	Bovine serum albumin
DDS	Drug delivery system
DDW	Double distilled water
DMA	2-(dimethylamino)ethyl methacrylate
FBS	Fetal bovine serum
GSH	Glutathione
HEMA	2-hydroxyethyl methacrylate
L-PD	Diol-conjugated fluorescent probe- B-PD
MC	Methylene chloride
MDCK	Madin-Darby Canine Kidney
MTT	3-(4,5-dimethylthiazol-2-yl)-2,5-diphenyltetrazolium bromide
NPC	Nitrophenyl chloroformate
PD	Polymer dots
PI	Propidium iodide
PTX	Paclitaxel
TEA	Triethylamine
