# Supplementary material for: Reduction-Triggered Paclitaxel Release Nano-Hybrid System Based on Core-Crosslinked Polymer Dots with a pH-Responsive Shell-Cleavable Colorimetric Biosensor
Source: Int J Mol Sci. 2019 Oct 28;20(21):5368. doi: 10.3390/ijms20215368 (PMC6862247; doi:10.3390/ijms20215368)
Supplement: Supplementary file 1 [file ijms-20-05368-s001.pdf]

Supplementary Materials

Reduction-Triggered Paclitaxel Release Nano-Hybrid System Based on Core-Crosslinked Polymer Dots with a pH-Responsive Shell-Cleavable Colorimetric Biosensor

Seul Gi Kim [1], Benny Ryplida [2], Pham Thi My Phuong [2], Hyun Jeong Won [1], Gibaek Lee [1], Suk Ho Bhang [3,*], and Sung Young Park [1,2,4,*]

[1] Department of Chemical and Biological Engineering, Korea National University of Transportation, Chungju 380-702, Korea; k.seulgi9797@gmail.com (S.G.K.); dnjsswh@ut.ac.kr (H.J.W.); glee@ut.ac.kr (G.L.)
[2] Department of Green Bio Engineering, Korea National University of Transportation, Chungju 380-702, Korea; ryplida@gmail.com (B.R.); ptmp.2411@gmail.com (P.T.M.P.)
[3] School of Chemical Engineering, Sungkyunkwan University, Suwon 16419, Korea
[4] Department of IT Convergence, Korea National University of Transportation, Chungju 380-702, Korea
* Correspondence: sukhobhang@skku.edu (S.H.B.); parkchem@ut.ac.kr (S.Y.P.); Tel.: +82-43-841-5225 (S.H.B.)

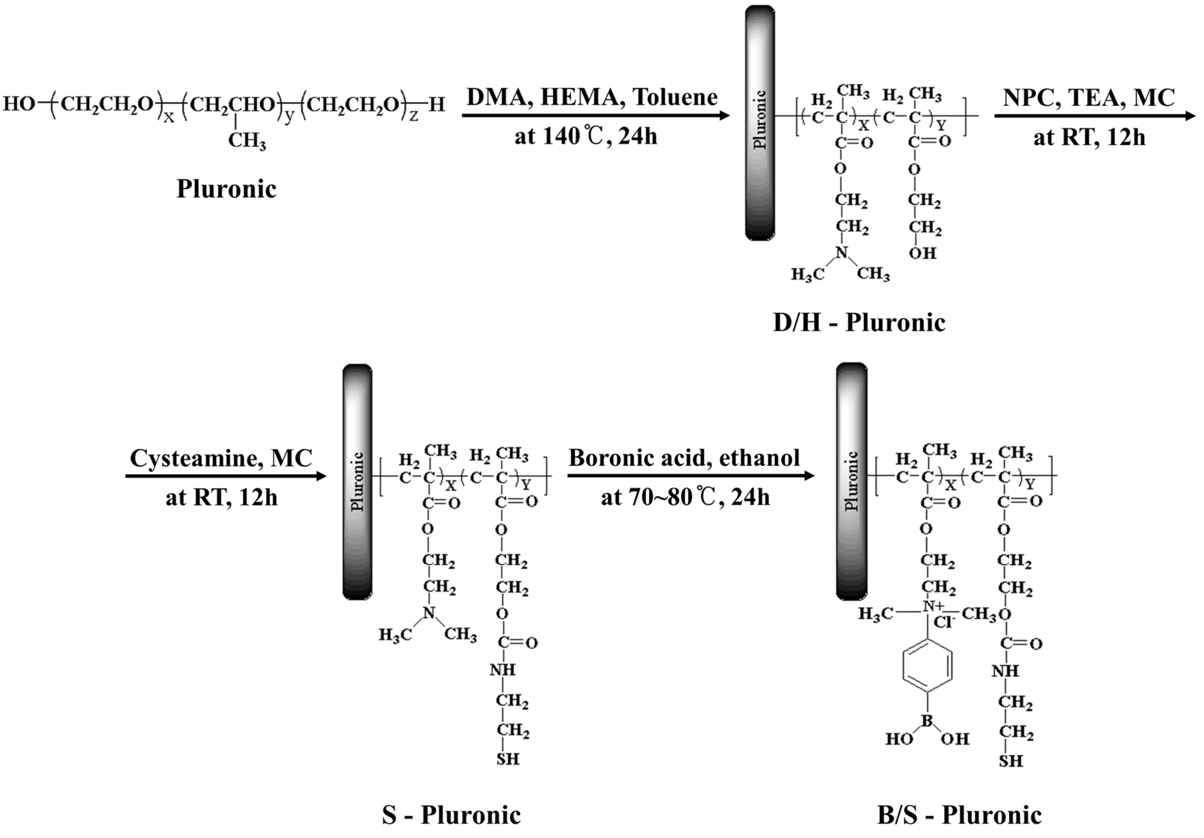

Scheme S1. Synthesis of B/S Pluronic.

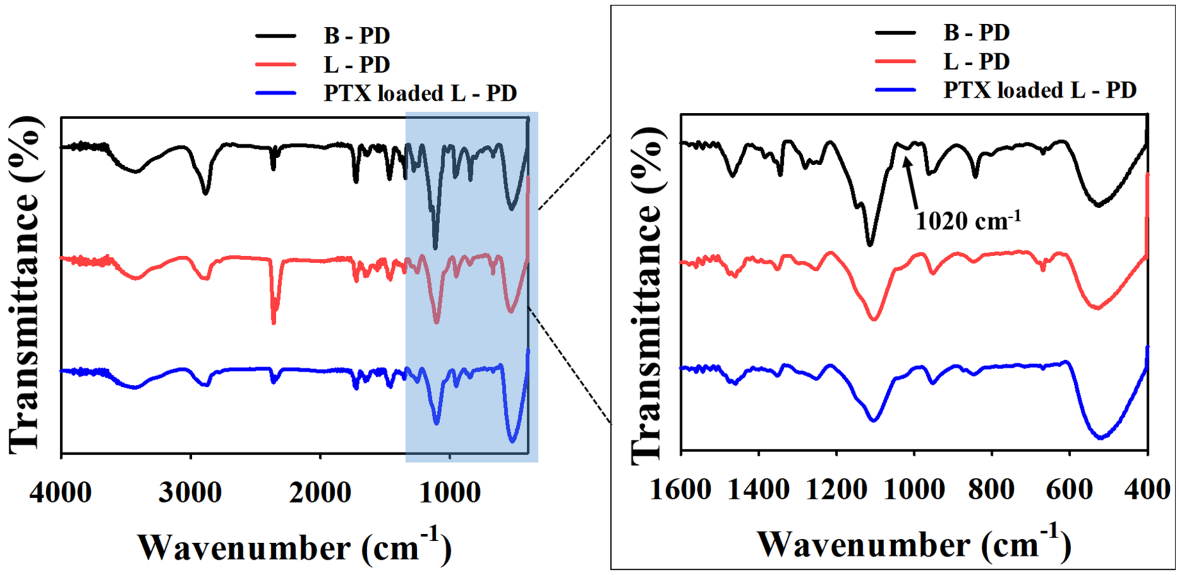

Figure S1. Fourier-transform Infrared (FT-IR) spectra of B-PD, L-PD, and PTX loaded L-PD.

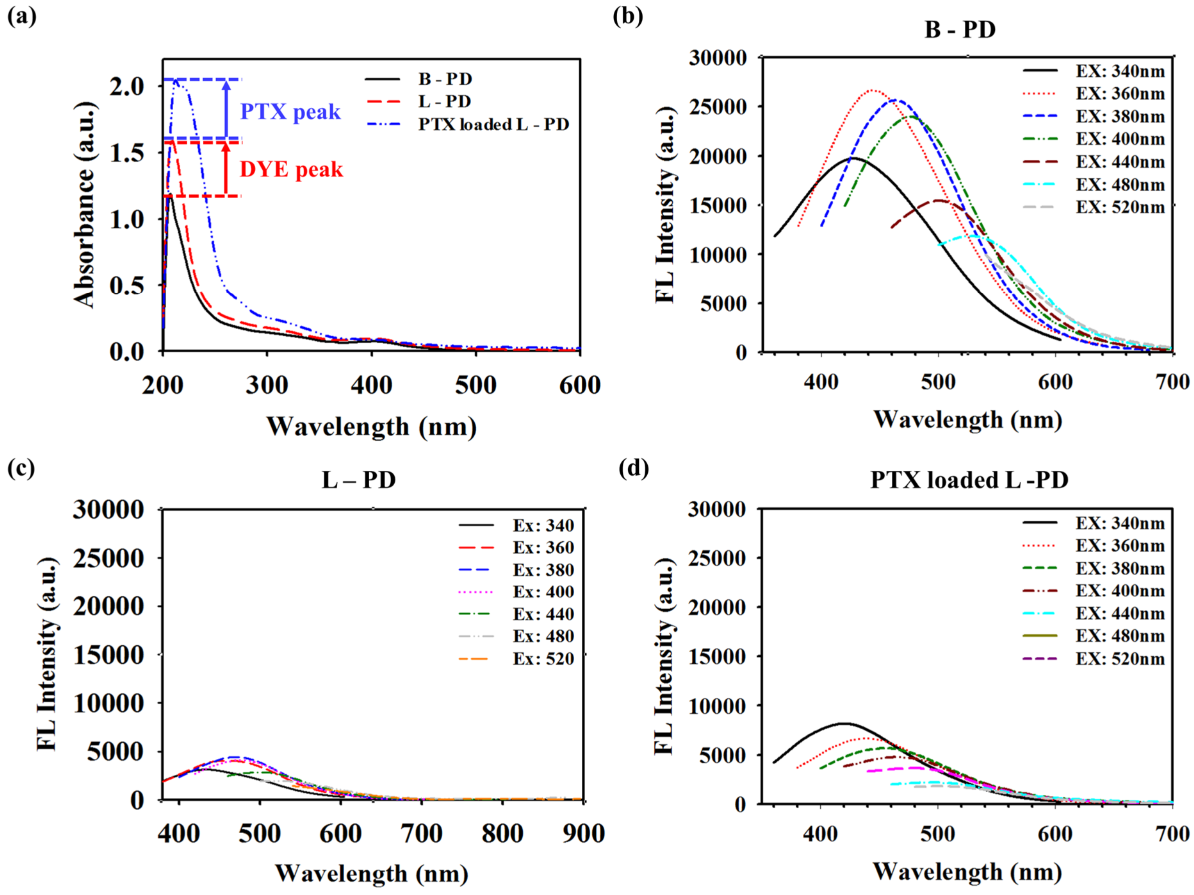

Figure S2. (**a**) UV–vis spectra of B-PD, L-PD, and PTX loaded L-PD (concentration 1 mg/mL). Photoluminescence spectra of (**b**) B-PD, (**c**) L-PD, and (**d**) PTX loaded L-PD.

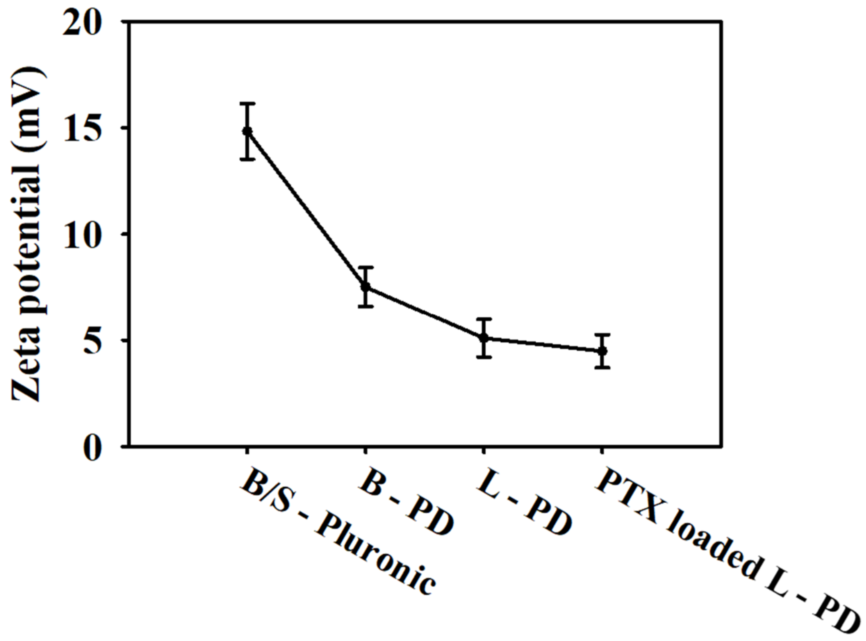

Figure S3. Zeta potentials of B/S-Pluronic, B-PD, L-PD, and PTX loaded L-PD in PBS pH 7.4 (n = 3).

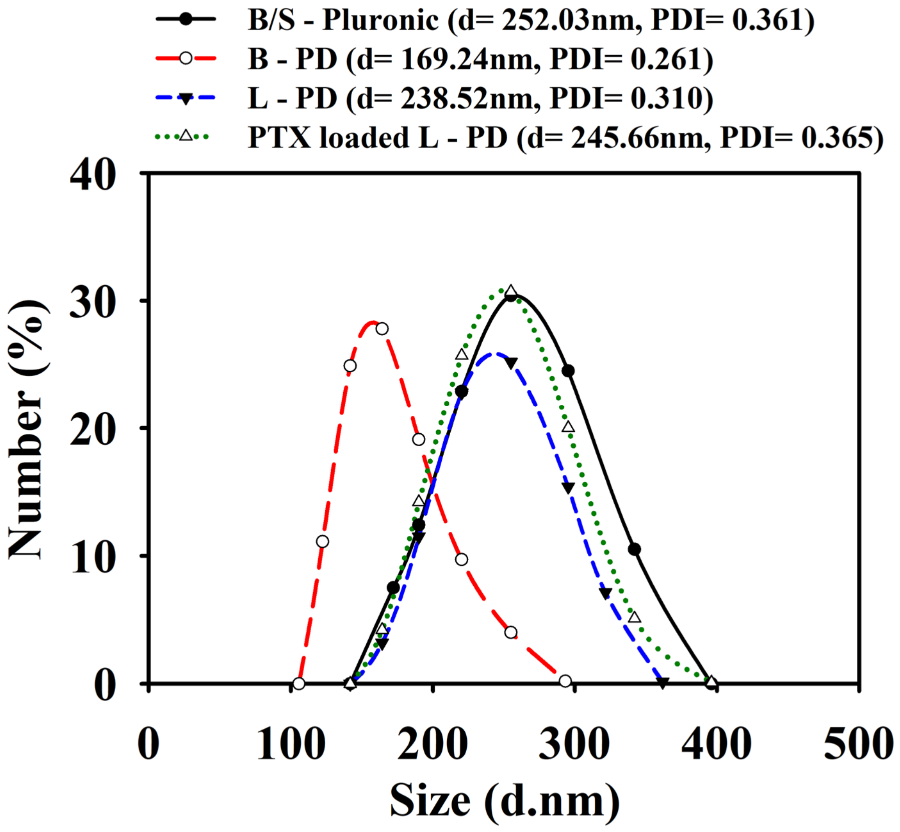

Figure S4. Average size distribution of of B/S-Pluronic, B-PD, L-PD, and PTX loaded L-PD in PBS solution pH 7.4 (Concentration: 0.5 mg/mL).

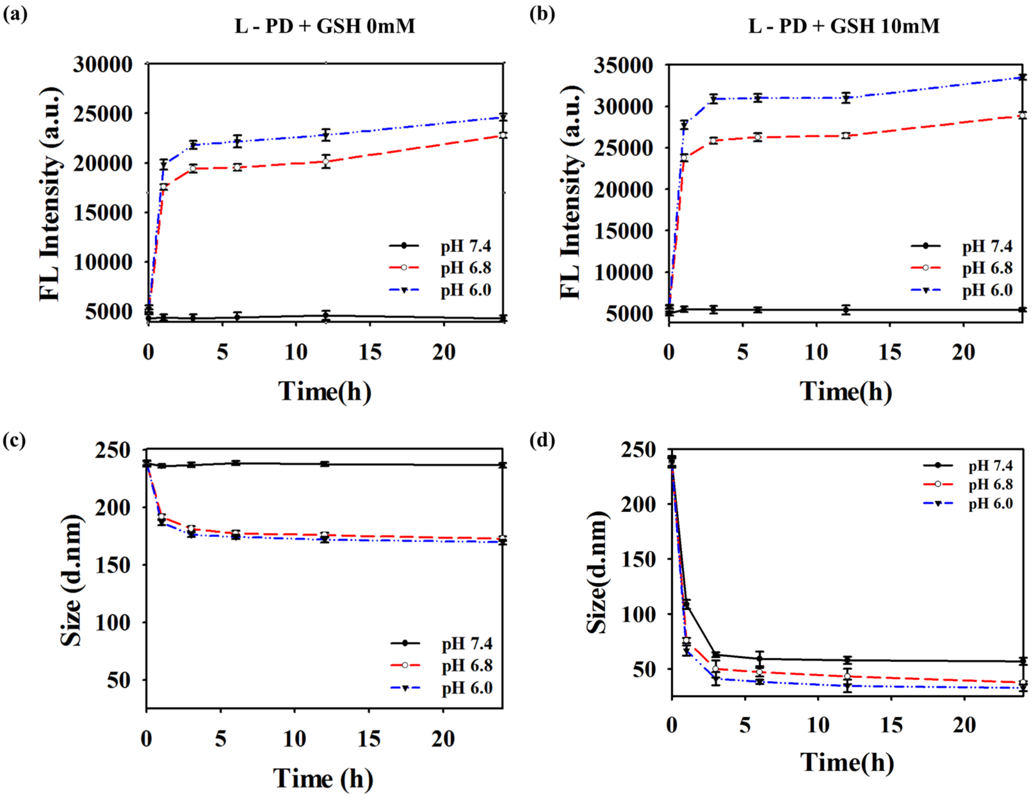

Figure S5. Luminescence intensity with excitation at λ = 360 nm and (**c**,**d**) DLS measurements of L-PD at different GSH concentrations (0 and 10 mm GSH) and pH values (6.0, 6.8, and 7.4) over time (n = 3).

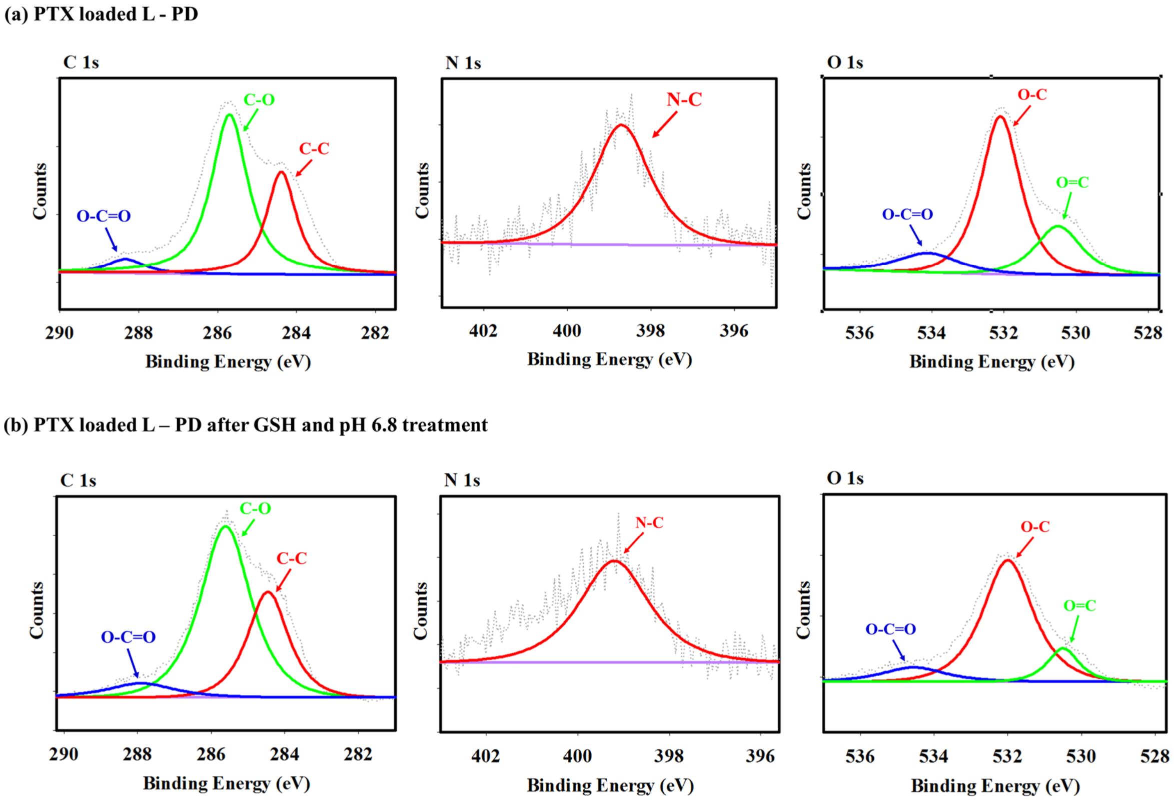

Figure S6. XPS narrow scan (C 1s, N 1s, and O 1s) of PTX loaded L-PD at pH 7.4 and 0 mM GSH (**a**) after treatment at pH 6.8 and 10 mM GSH (**b**).
